# Validity of the SARC-F questionnaire in assessing sarcopenia in patients with chronic kidney disease: a cross-sectional study

**DOI:** 10.3389/fmed.2023.1188971

**Published:** 2023-07-18

**Authors:** Wen Du, Chenni Gao, Xuejie Wang, Xiaobo Ma, Jingyuan Xie, Haijin Yu, Zhenhua Yang, Zijin Chen, Xiaonong Chen

**Affiliations:** ^1^Department of Nephrology, Ruijin Hospital, Shanghai Jiao Tong University School of Medicine, Shanghai, China; ^2^Department of Nephrology, RuiJin Hospital LuWan Branch, Shanghai Jiaotong University School of Medicine, Shanghai, China; ^3^Department of Nephrology, Wuxi Branch of Ruijin Hospital, Wuxi, Jiangsu Province, China

**Keywords:** chronic kidney disease, sarcopenia, screening, maintenance hemodialysis, SARC-F

## Abstract

**Objective:**

To examine the validity of the 5-component SARC-F questionnaire for screening sarcopenia among patients with chronic kidney disease (CKD).

**Methods:**

Eligible participants were enrolled from the Department of Nephrology, Ruijin Hospital, Shanghai Jiao Tong University School of Medicine from March 2019 to November 2019. Evaluations were performed using the self-administered SARC-F questionnaire. Sarcopenia was diagnosed by grip strength, the chair stand test and appendicular skeletal muscle mass. The severity of sarcopenia was evaluated by gait speed. We calculated the sensitivity and specificity of the SARC-F to evaluate construct validity. Moreover, receiver operating characteristic (ROC) curve analysis was performed to identify the cutoff value for nondialysis-dependent (NDD) CKD patients’ and maintenance hemodialysis (MHD) patients’ scores.

**Results:**

A total of 105 NDD-CKD patients and 125 MHD patients were included, and the prevalence of sarcopenia was 5.7 and 31.2%, respectively. Among them, there were 21 (16.8%) MHD patients with severe sarcopenia but no NDD-CKD patients with severe sarcopenia. The sensitivity and specificity of the SARC-F were 16.7 and 98.0% for NDD-CKD patients, and 48.7 and 89.5% for MHD patients, respectively. For NDD-CKD patients, the area under the receiver operating characteristic curve (AUROC) of the total SARC-F score was 0.978 (95% confidence interval (CI): 0.929–0.997, *p* < 0.001), and the cutoff value of 1 reached the highest Youden index of 0.950 and max ROC curve area of 0.974. For MHD patients, the AUROC of the total SARC-F score was 0.730 (95% CI: 0.644–0.806, *p* < 0.001), and the cutoff value of 4 reached the highest Youden index of 0.383 and max ROC curve area of 0.691.

**Conclusion:**

CKD patients, especially MHD patients, were at high risk of suffering sarcopenia. The SARC-F had low-to-moderate sensitivity but high specificity for screening sarcopenia among patients with CKD. The best cutoff values of the SARC-F score were different for screening sarcopenia among NDD-CKD and MHD patients.

## Introduction

Sarcopenia, defined as a skeletal muscle wasting syndrome characterized by decreased skeletal muscle mass, decreased skeletal muscle strength, and decreased physical activity with increasing age (1), affects not only older people but also patients with diseases such as cancer, chronic kidney disease (CKD), or with malnutrition. The definition of sarcopenia was originally proposed in 2010 by the European Working Group on Sarcopenia in Older People (EWGSOP) and was updated in January 2019 as EWGSOP2 (2).

Sarcopenia increases the risk of falls and fractures, reduces quality of life, and increases mortality (3). However, the current understanding of sarcopenia is insufficient, and sarcopenia is easy to ignore. Thus, it is important for practicing nephrologists to identify and manage sarcopenia early.

Clinical tools, such as anthropometric measures (i.e., dual-energy X-ray absorptiometry (DXA) or the hand grip and chair stand tests) or physical performance tests (i.e., the 4-m walk test), aimed at evaluating muscle strength and muscle mass (4), are feasible but relatively complicated. Simple, secure, and inexpensive screening tools with good performance would be convenient and helpful for medical staff. In 2012, Malmstrom and Morley created and validated the SARC-F, which is a self-administered questionnaire that assesses five items: strength, assistance in walking, rising from a chair, climbing stairs and falls. Each evaluation item is scored according to the degree of difficulty or frequency difference, and a person whose score ≥ 4 points is suspected of having sarcopenia clinically (5). The EWGSOP2 recommended the use of the SARC-F questionnaire (SARC-F) to find sarcopenia-associated symptoms quickly.

The validity of the SARC-F for patients with CKD has not yet been fully examined. Therefore, the objective of this study was to examine the validity of the SARC-F among patients with (NDD-CKD) and maintenance hemodialysis (MHD). We conducted a cross-sectional study to investigate the clinical value of the SARC-F scale in the screening of sarcopenia among NDD-CKD and MHD patients.

## Methods

### Participants and study design

Participants included in this study were treated at the Department of Nephrology, Ruijin Hospital, Shanghai Jiao Tong University School of Medicine from March 2019 to November 2019. We enrolled both NDD-CKD patients and MHD patients consecutively. For NDD-CKD patients, the inclusion criteria included chronic kidney structural or functional abnormalities due to various causes ≥3 months, including abnormalities in urine, histology, imaging, or unexplained estimated glomerular filtration rate (eGFR) <60 mL/min/1.73 m^2^. For MHD patients, the inclusion criteria included hemodialysis duration ≥3 months. For both NDD-CKD and MHD patients, the exclusion criteria were age < 18 and > 80 years, acute kidney injury, severe mental illness, limb deformity or movement disorder, pregnancy or lactation, diagnosis of heart failure or severe infection within the past 6 months, active cancer or liver disease at the time of evaluation, and inability or unwillingness to participate in the survey.

As for the simple size, first, we calculated margin of error in NDD-CKD and MHD patients. Then, we calculated the simple size using the formula:

$n=p\times \left(1–p\right)\times {\left(z÷E\right)}^{2}$

*n* means the minimal number of necessary samples, *p* means the population proportion, *z* is the confidence level of the interval, and *E* is the margin of error we calculated before. The patients enrolled in our study achieved the minimum sample size required.

This study was approved by the ethics committee of the hospital (1.0/20200201), and informed consent was obtained from each patient.

### The SARC-F questionnaire

A specific researcher was responsible for evaluating all participants using the SARC-F. Participants were provided with three possible answers for each item: “no problem,” “some problems,” and “many problems or impossible.” For falls, the possible answers were “never,” “one to three times,” and “four times or more.” Each level was scored with 0, 1, and 2 points, respectively. The patients answered the questionnaire with the help of the specific researcher. The questionnaire was translated from English to Chinese in comprehensive language by the researchers in our department. Most participants included were able to read. For those who had difficulties in reading, the researcher explained the questionnaire to his/her next of kin, and the questionnaire was finished by his/her next of kin. The total score ranged from 0 to 10, with scores of ≥4 points indicating the risk of sarcopenia (6).

### Muscle strength measurements

Hand grip strength (HGS) and the chair stand test are the gold standards for muscle strength measurement (7). In our study, HGS was assessed using a dynamometer (Jamar Hydraulic Hand Evaluation Kit, United States). Patients were first familiarized with the device and were then seated with shoulder adduction in line with the trunk, elbow bent 90°, the forearm and wrist in a neutral position, and the proximal metacarpophalangeal joint bent approximately 90°. Patients were instructed to grip the dynamometer with maximum strength in response to a voice command, and the highest value of three measurements was considered for this study. Nonfistula hands were tested among MHD patients, and dominant hands were tested among NDD-CKD patients. The chair stand test measured the amount of time needed for a patient to stand and sit five times with arms crossed as quickly as possible (8).

### Measurement of muscle mass

Dual-energy X-ray absorptiometry (DXA) is considered an accurate and reproducible method to evaluate body composition in clinical practice (9). In our study, DXA (Lunar X-ray bone densitometer, version: LU43616ZH-CN) was used by a trained technician to measure appendicular muscle mass (ASM). ASM was calculated as the sum of muscle mass (in kilograms) in all 4 limbs and was correlated with body size. We calculated the appendicular muscle mass index (ASMI) for each participant as the absolute level of ASM adjusted for height squared (ASM/height^2^). The machine was regularly calibrated.

### Assessment of physical performance

The 4-min usual walking speed test was used to measure gait speed in this study (10). Timing began when the command was given; the time, in seconds, needed to complete the walk was recorded.

### Diagnosis of sarcopenia

The diagnosis of sarcopenia was based on the presence of derangements in both muscle strength and muscle mass. Based on the diagnostic criteria for sarcopenia established by EWGSOP2 (2), meeting the following 2 criteria can be diagnosed as sarcopenia: (i) HGS < 27 kg for men and < 16 kg for women and/or the chair stand test >15 s for five rises; and (ii) ASMI <7.0 kg/m2 for men and < 5.5 kg/m2 for women. We also evaluated the severity of sarcopenia using the definition of severe sarcopenia with gait speed ≤0.8 m/s.

### Statistical analysis

Data with a normal distribution are described as the means ± standard deviations, and data with a nonnormal distribution are described as the medians (interquartile ranges). Categorical data were expressed as numbers of cases (percentages). An independent sample *t* test was used to compare the differences between groups with normally distributed data. For data with nonnormal distributions, the Mann–Whitney *U* test was applied. The chi-square test was used to compare the differences between groups for categorical data. To evaluate construct validity, sensitivity, specificity and predictive values of the SARC-F with sarcopenia, the diagnostic criteria of EWGSOP2 as the reference were calculated. Moreover, a receiver operating characteristic (ROC) curve analysis was performed to identify the cutoff value for NDD-CKD patients’ and MHD patients’ scores. Body mass index (BMI) was calculated as weight (in kilograms) divided by the square of height (in meters). The concordance between the SARC-F scores and the EWGSOP2 criteria classifications was determined using Cohen’s kappa coefficients and proportion of agreement. The concordance was defined as poor (0–0.20), fair (0.21–0.40), moderate (0.41–0.60), good (0.61–0.80), and optimal (0.81–1). The reclassification percentage was determined as 100% agreement. All statistical analyses were performed using STATA version 15.0, and a *p* value ≤0.05 was considered indicative of statistical significance.

## Results

### Clinical characteristics of NDD-CKD and MHD patients

This study included one hundred five NDD-CKD patients and one hundred twenty-five MHD patients. The mean age of NDD-CKD patients was 52.1 years, while that of MHD patients was 59.4 years. Regarding the cause of kidney disease, 75.2% of NDD-CKD patients had chronic glomerulonephritis, while 48.4% of MHD patients had chronic glomerulonephritis. The clinical characteristics, complications, use of medications, and distribution of SARC-F scores for each group are shown in Table 1. Compared to NDD-CKD patients, MHD patients were older, with higher SARC-F scores and lower BMI (24.4 ± 3.5 vs. 22.0 ± 3.7 kg/m^2^, *p* < 0.001), ASM (18.7 ± 4.2 vs. 16.9 ± 4.2 kg, *p* = 0.002), ASMI (6.7 ± 1.0 vs. 6.2 ± 1.1 kg/m^2^, *p* < 0.001), and HGS (31.6 ± 11.2 vs. 22.6 ± 11.2 kg, *p* < 0.001).

**Table 1:** Comparison of clinical features of patients in the NDD-CKD and MHD groups.

	NDD-CKD						MHD	*P*
	Stage 1	Stage 2	Stage 3	Stage 4	Stage 5	Overall		
N, n	20	23	24	19	19	105	125	
Age (years)	45.9 ± 8.6	54.6 ± 8.7	49.0 ± 10.7	55.3 ± 9.5	56.4 ± 13.9	52.1 ± 11.0	59.4 ± 14.9	<0.001*
Males [n(%)]	8 (40.0)	13 (56.5)	9 (37.5)	15 (78.9)	7 (36.8)	52 (49.5)	68 (54.4)	0.461
BMI (kg/m^2^)	25.0 ± 3.7	24.6 ± 3.5	24.9 ± 3.2	25.1 ± 2.9	22.2 ± 3.4	24.4 ± 3.5	22.0 ± 3.7	<0.001*
Chronic glomerulonephritis [n(%)]	18 (90.0)	20 (87.0)	19 (79.2)	12 (63.2)	10 (52.6)	79 (75.2)	61 (48.8)	<0.001*
Dialysis vintage (months)	–	–	–	–	–	–	69.3 ± 58.1	–
Comorbidities [n(%)]
Hypertension	7 (35.0)	13 (56.5)	16 (66.7)	16 (84.2)	14 (73.7)	66 (62.9)	109 (87.2)	<0.001*
Diabetes	1 (5.0)	7 (30.4)	3 (12.5)	5 (26.3)	3 (15.8)	19 (18.1)	32 (25.6)	0.172
Cardiovascular disease	0 (0)	1 (4.3)	3 (12.5)	3 (15.8)	1 (5.3)	8 (7.6)	49 (39.2)	<0.001*
History of joint replacement	0 (0)	0 (0)	0 (0)	0 (0)	0 (0)	0 (0)	2 (1.6)	<0.001*
Medical history
Use of steroids	1 (5.0)	1 (4.3)	2 (8.3)	5 (26.3)	3 (15.8)	12 (11.4)	3 (2.4)	0.007*
Smoking	1 (5.0)	9 (39.1)	3 (12.5)	2 (10.5)	3 (15.8)	18 (17.1)	32 (25.6)	0.121
Alcohol consumption	2 (10.0)	6 (26.1)	0 (0.0)	1 (5.3)	1 (5.3)	10 (9.5)	31 (24.8)	0.003*
SARC-F score	0 (0, 0)	0 (0, 0)	0 (0, 0)	0 (0, 0)	0 (0, 1)	0 (0, 0)	1 (0, 4)	<0.001*
ASM (kg)	19.3 ± 4.5	18.8 ± 3.9	18.1 ± 4.2	20.2 ± 3.7	17.1 ± 4.6	18.7 ± 4.2	16.9 ± 4.2	0.002*
ASMI (kg/m^2^)	6.9 ± 1.1	6.7 ± 1.0	6.6 ± 1.0	7.1 ± 0.9	8.2 ± 1.1	6.7 ± 1.0	6.2 ± 1.1	<0.001*
HGS (kg)	35.5 ± 11.6	34.1 ± 11.3	30.2 ± 9.6	34.2 ± 10.3	23.7 ± 10.4	31.6 ± 11.2	22.6 ± 11.2	<0.001*
Chair stand (s)	9.2 (7.5, 10.7)	10.1 (9.0, 12.1)	10.6 (8.8, 11.7)	9.8 (8.3, 10.7)	10.2 (7.6, 13.5)	9.9 (8.6, 11.6)	11.0 (8.6, 13.0)	0.002*
Gait speed (m/s)	1.19 (1.11, 1.34)	1.12 (1.02, 1.25)	1.12 (1.01, 1.29)	1.14 (1.03, 1.24)	1.15 (0.97, 1.29)	1.15 (1.03, 1.28)	1.04 (0.87, 1.25)	0.001*

*P < 0.05. The P value is for the comparison of MHD patients and CKD-NDD patients.

CKD, chronic kidney disease with nondialysis dependence; MHD, maintenance hemodialysis; BMI, body mass index; HGS, handgrip strength; ASM, appendicular skeletal muscle mass; ASMI, appendicular skeletal muscle mass index.

Compared with nonsarcopenic patients, sarcopenic patients had a lower BMI in both the NDD-CKD (24.8 ± 3.2 vs. 20.1 ± 1.1 kg/m^2^, *p* < 0.001) and MHD groups (22.7 ± 3.8 vs. 20.4 ± 2.9 kg/m^2^, *p* = 0.001). Moreover, sarcopenic patients in the MHD group were older (66.82 ± 11.69 vs. 56.01 ± 14.99 years, *p* < 0.001) and had a lower gait speed than nonsarcopenic patients. The comparison of clinical characteristics and of the analyzed patients between the sarcopenia and non-sarcopenia groups according to the EWGSOP2 guidelines are shown in Table 2.

**Table 2:** Clinical characteristics of patients with sarcopenia and nonsarcopenia according to the EWGSOP2 guidelines.

	NDD-CKD			MHD		
	Sarcopenia (*n* = 6)	Nonsarcopenia (*n* = 99)	*P*	Sarcopenia (*n* = 39)	Nonsarcopenia (*n* = 86)	*P*
Age (years)	52.70 ± 18.7	52.07 ± 10.33	0.944	66.82 ± 11.69	56.01 ± 14.99	<0.001*
Males [n(%)]	3 (50.0)	49 (49.5)	0.981	23 (59.0)	45 (52.3)	0.489
BMI (kg/m^2^)	20.1 ± 1.1	24.8 ± 3.2	<0.001*	20.4 ± 2.9	22.7 ± 3.8	0.001*
Comorbidities [n(%)]
Hypertension	2 (33.3)	64 (64.6)	0.123	35 (89.7)	74 (86.0)	0.566
Diabetes	1 (16.7)	18 (18.2)	0.925	14 (35.9)	18 (20.9)	0.076
Cardiovascular disease	0 (0)	8 (8.1)	0.469	19 (48.7)	30 (34.9)	0.142
Dialysis vintage (months)	/	/	/	76.9 ± 69.2	65.8 ± 52.3	0.375
SARC-F score	2 (1, 3.75)	0 (0, 0)	<0.001*	3 (1, 5)	0 (0, 2)	<0.001*
ASM (kg)	14.79 ± 4.16	18.90 ± 4.14	0.020*	14.51 ± 3.08	17.93 ± 4.17	<0.001*
ASMI (kg/m^2^)	5.41 ± 0.85	6.78 ± 1.00	0.001*	5.41 ± 0.87	6.56 ± 1.07	<0.001*
HGS (kg)	16.8 ± 4.1	32.5 ± 10.9	<0.001*	13.6 ± 6.9	26.6 ± 10.4	<0.001*
Chair stand (s)	13.5 (12.5, 15.1)	9.8 (7.3, 11.0)	<0.001*	13.7 (12.0, 19.1)	10.2 (7.9, 12.3)	<0.001*
Gait speed (m/s)	1.06 (0.89, 1.18)	1.12 (1.03, 1.25)	0.139	0.80 (0.46, 1.02)	1.02 (0.86, 1.19)	<0.001*

*P < 0.05.

CKD-NDD, chronic kidney disease with nondialysis dependence; MHD, maintenance hemodialysis; BMI, body mass index; HGS, handgrip strength; ASM, appendicular skeletal muscle mass; ASMI, appendicular skeletal muscle mass index.

### The incidence of sarcopenia according to the EWGSOP2 criteria

Six NDD-CKD patients (5.7%) and thirty-nine MHD patients (31.2%) were diagnosed with sarcopenia according to the diagnostic criteria of EWGSOP2. In addition, there was no severe sarcopenia among NDD-CKD patients, and twenty-one MHD patients were diagnosed with severe sarcopenia. The presence or absence and distribution of sarcopenia based on the diagnostic criteria of EWGSOP2 and SARC-F are shown in Table 3.

**Table 3:** Distribution of SARC-F scores of NDD-CKD and MHD patients.

	NDD-CKD SARC-F, frequency (%)		MHD SARC-F, frequency (%)	
	Nonsarcopenia (*n* = 99)	Sarcopenia (*n* = 6)	Nonsarcopenia (*n* = 86)	Sarcopenia (*n* = 39)
Strength
No difficulty	94 (94.9)	1 (16.7)	60 (69.8)	11 (28.2)
Some difficulty	5 (5.1)	5 (83.3)	18 (20.9)	16 (41.0)
Extreme difficulty or inability	0 (0)	0 (0)	8 (9.3)	12 (30.8)
Assistance in walking
No difficulty	98 (99.0)	5 (83.3)	76 (88.4)	27 (69.2)
Some difficulty	1 (1.0)	1 (16.7)	5 (5.8)	11 (28.2)
Extreme difficulty or inability	0 (0)	0 (0)	5 (5.8)	1 (2.6)
Rising from a chair
No difficulty	98 (99.0)	4 (66.7)	69 (80.2)	20 (51.3)
Some difficulty	1 (1.0)	1 (16.7)	13 (15.1)	14 (35.9)
Extreme difficulty or inability	0 (0)	1 (16.7)	4 (4.7)	5 (12.8)
Climbing stairs
No difficulty	97 (98.0)	2 (33.3)	63 (73.3)	15 (38.4)
Some difficulty	2 (2.0)	3 (50.0)	17 (19.8)	12 (30.8)
Extreme difficulty or inability	0 (0)	1 (16.7)	6 (6.9)	12 (30.8)
Falls
None	98 (99.0)	5 (83.3)	78 (90.7)	35 (89.7)
1–3	1 (1.0)	1 (16.7)	8 (9.3)	3 (7.7)
≥4	0 (0)	0 (0)	0 (0)	1 (2.6)

CKD-NDD, chronic kidney disease with nondialysis dependence; MHD, maintenance hemodialysis.

### The concordance between the SARC-F scores and the EWGSOP2 criteria

**Figure 1:**
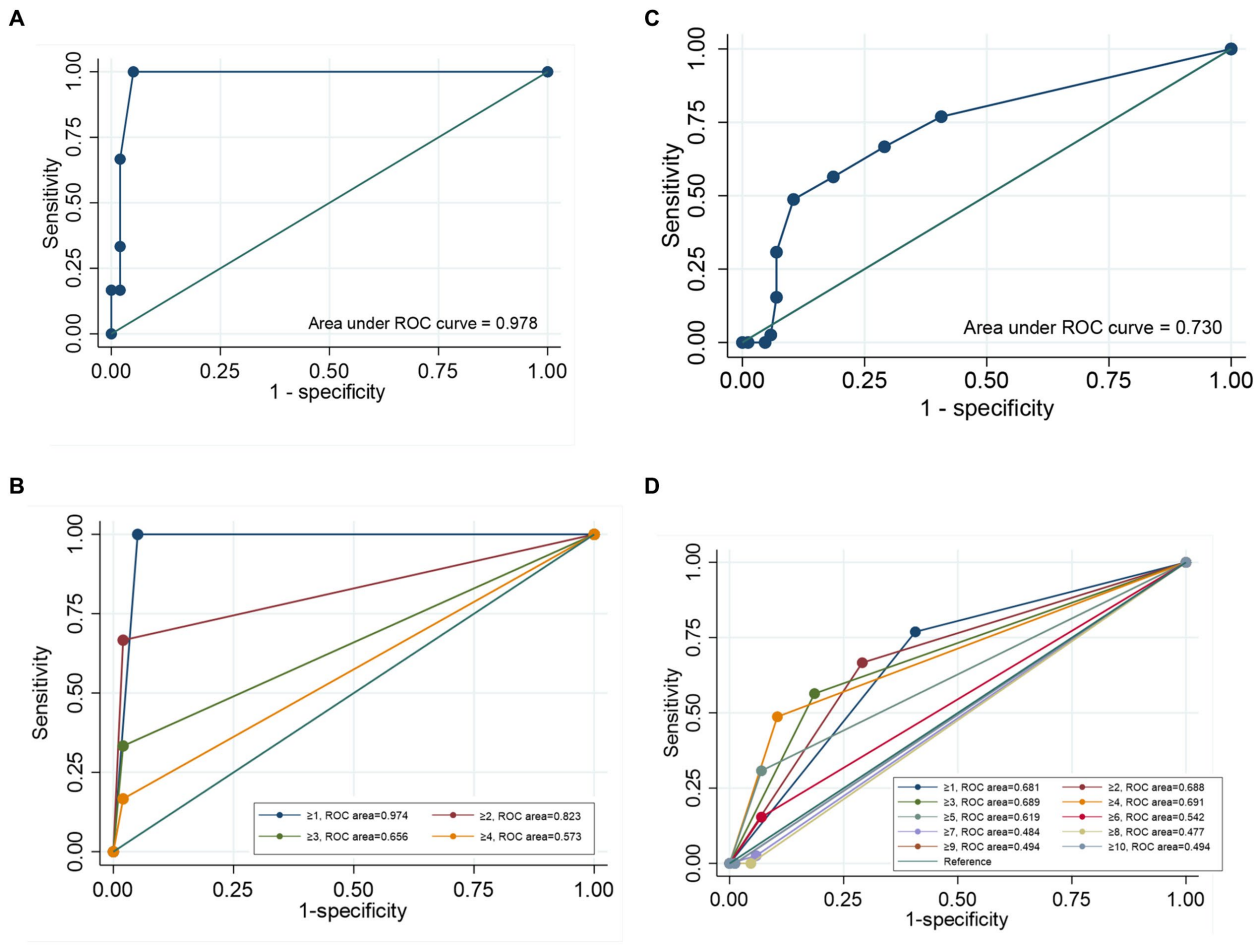
Receiver operating characteristic (ROC) curve of the SARC-F score for screening sarcopenia. **(A)** The ROC curve of the SARC-F score for screening sarcopenia in NDD-CKD patients. **(B)** The ROC curve for screening sarcopenia at each cutoff point for NDD-CKD patients. **(C)** The ROC of the SARC-F score for screening sarcopenia in MHD patients. **(D)** The ROC curve for screening sarcopenia at each cutoff point for MHD patients.

The sensitivity and specificity of the SARC-F were 16.7 and 98.0% for NDD-CKD patients, and 48.7 and 89.5% for MHD patients, respectively. The positive and negative predictive value was 33.3 and 95.1% for NDD-CKD patients, and 67.9 and 79.4% for MHD patients, respectively. Concordance between the SARC-F algorithm and the EWGSOP2 algorithm for NDD-CKD patients was poor, with a kappa coefficient (κ) of 0.19 and a reclassification rate of 6.7% (Table 4). The area under the receiver operating characteristic curve (AUROC) of the total SARC-F score was 0.978 (95% confidence interval (CI): 0.929–0.997, *p* < 0.001) (Figure 1A), and the cutoff value of 1 reached the highest Youden index of 0.950 and max ROC curve area of 0.974, with sensitivity of 100.00% and specificity of 94.50% (Supplementary Table 1 and Figure 1B).

**Table 4:** Reclassification table of sarcopenia incidence using the SARC-F and the EWGSOP2 guidelines.

	NDD-CKD SARC-F, frequency (%)		MHD SARC-F, frequency (%)	
	Sarcopenia	Nonsarcopenia	Sarcopenia	Nonsarcopenia
EWGSOP2
Sarcopenia	1 (0.9)	5 (4.8)	19 (15.2)	20 (16.0)
Nonsarcopenia	2 (1.9)	97 (92.4)	9 (7.2)	77 (61.6)

The concordance between the SARC-F algorithm and the EWGSOP2 algorithm was poor (*κ* = 0.19), with a reclassification rate of 6.7%.

In contrast, 20 (16.0%) subjects without sarcopenia according to the SARC-F algorithm were diagnosed with sarcopenia following the EWGSOP2 algorithm. A κ of 0.11 with a reclassification rate of 56.0% demonstrated obvious discrepancies between the SARC-F algorithm and the EWGSOP2 algorithm (Table 4). The AUROC of the total SARC-F score was 0.730 (95% CI: 0.644–0.806, *p* < 0.001) (Figure 1C), and the cutoff value of 4 reached the highest Youden index of 0.383 and max ROC curve area of 0.691, with a sensitivity of 48.7% and specificity of 89.5% (Supplementary Table 1 and Figure 1D).

## Discussion

In our study, the prevalence of sarcopenia was 5.7% among NDD-CKD patients and 31.2% among MHD patients. For patients with CKD, especially CKD stages 3–5, the decrease in skeletal muscle mass is significantly correlated with the decline in renal function. Deterioration in renal function, long-term dialysis, the uremic milieu, and reduced activity will accelerate the decline in skeletal muscle strength and the decrease in skeletal muscle mass, making patients more likely to suffer from sarcopenia (11, 12). Moon et al. (13) reported the increased prevalence of sarcopenia with increased CKD stage: 2.6% among normal and CKD stages 1–2 patients and 5.6% among CKD stages 3–5 patients. Among maintenance hemodialysis (MHD) patients, the prevalence of sarcopenia has been reported to be between 16 and 40% (14–16).

Sarcopenia is associated with adverse health outcomes such as depression, falls, fracture, cognitive impairment, and even mortality (16–18). The SARC-F questionnaire has been suggested to be one of the best simplified screening tools for sarcopenia in primary care (2, 19). Thus, we aimed to examine the validity of the SARC-F for screening sarcopenia among patients with NDD-CKD along with MHD patients.

In the present study, the SARC-F had a sensitivity of 16.7% and a specificity of 98.0% for NDD-CKD patients. However, compared to a value of 4, the cutoff value of 1 reached the optimal Youden index for NDD-CKD patients, with 100% sensitivity and 94.8% specificity. However, for MHD patients, SARC-F scores ≥4 reached the optimal Youden index, but the SARC-F only had a low-to-moderate specificity of 48.7% and higher sensitivity of 89.5%.

Thus, the screening value of the SARC-F is relatively satisfactory for NDD-CKD patients but limited for MHD patients. These features make it a practical tool for screening sarcopenia among NDD-CKD patients. Compared with muscle mass and strength evaluations, the use of the SARC-F can reduce time and cost through its superior ability to identify patients highly likely to have sarcopenia. According to our study, we suggested that the cutoff value of 1 was appropriate for NDD-CKD patients, and patients with a SARC-F score ≥ 1 should be examined for the occurrence of sarcopenia early.

Recently, a study enrolled 179 MHD patients (mean age 66.5 ± 12 years) using the Asian Working Group for Sarcopenia (AWGS2019) for the diagnostic criteria and showed that the sensitivity and specificity values of the SARC-*F* ≥ 4 were 42.9 and 70.8%, respectively (16). The possible reason for the different accuracies of the SARC-F may be due to the different age distributions and diagnostic criteria of the two studies. However, because of the relatively limited sensitivity and specificity of the SARC-F for MHD patients, the SARC-F is not practical for screening sarcopenia in MHD patients alone and should be combined with other measurements, such as muscle mass and physical performance.

Our study has several limitations. First, the cross-sectional analysis prevented us from establishing the causes and effects between sarcopenia and poor physical performance. Second, our analysis should be interpreted with caution because of the small number of participants defined as being sarcopenic using the EWGSOP2 criteria, and multicenter studies are indeed needed for further exploration. In addition, we removed the person who could not finish the muscle strength or 4-min usual walking speed test, which may cause selection bias.

This is the first study to assess the validity of the SARC-F among patients with both NDD-CKD and MHD. Our study revealed that the prevalence of sarcopenia was high in CKD, especially among MHD patients, demanding the attention of clinical staff. In summary, the SARC-F might be used as a screening and diagnostic tool with different cutoff values for sarcopenia among CKD patients in clinical practice.

## Practical application

Patients with CKD, especially those with MHD, were at high risk of suffering sarcopenia. The SARC-F questionnaire is a simplified, convenient and feasible screening tool for sarcopenia. NDD-CKD patients with a SARC-F score ≥ 1 should be examined for the occurrence of sarcopenia. For MHD patients, the SARC-F is not practical enough and should be combined with other measurements.

## Data availability statement

The original contributions presented in the study are included in the article/Supplementary material, further inquiries can be directed to the corresponding authors.

## Ethics statement

The studies involving human participants were reviewed and approved by the Ethics Committee of Ruijin Hospital, Shanghai Jiaotong University, School of Medicine. The patients/participants provided their written informed consent to participate in this study.

## Author contributions

WD: conceptualization, methodology, investigation, formal analysis, and writing—original draft. CG: formal analysis, data curation, and writing—review and editing. XW: investigation, conceptualization, methodology, and data curation. XM: conceptualization, methodology, and data curation. JX, HY, and ZY: investigation and formal analysis. ZC: conceptualization, methodology, data curation, project administration, and writing—review and editing. XC: project administration and funding acquisition. All authors contributed to the article and approved the submitted version.

## Funding

This work was supported by the Key Clinical Specialty Construction Project of Shanghai (grant number: shslczdzk02502), the National Natural Science Foundation of China (81600590), Shanghai Sailing Program (20YF1428400), Shanghai Health Commission Scientific Research Project (20204Y0126), and Zhongguancun Nephrology and Blood Purification Innovation Alliance Youth Research Fund (grant number: NBPIA20QC0302).

## Conflict of interest

JX declared that they were an editorial board member of Frontiers, at the time of submission. This had no impact on the peer review process and the final decision.

The remaining authors declare that the research was conducted in the absence of any commercial or financial relationships that could be construed as a potential conflict of interest.

## Publisher’s note

All claims expressed in this article are solely those of the authors and do not necessarily represent those of their affiliated organizations, or those of the publisher, the editors and the reviewers. Any product that may be evaluated in this article, or claim that may be made by its manufacturer, is not guaranteed or endorsed by the publisher.
